# Mass Spectrometry-Based Method of Detecting and Distinguishing Type 1 and Type 2 Shiga-Like Toxins in Human Serum

**DOI:** 10.3390/toxins7124875

**Published:** 2015-12-02

**Authors:** Christopher J. Silva, Melissa L. Erickson-Beltran, Craig B. Skinner, Stephanie A. Patfield, Xiaohua He

**Affiliations:** Western Regional Research Center, United States Department of Agriculture, Albany, CA 94710, USA; christopher.silva@ars.usda.gov (C.J.S.); melissa.erickson@ars.usda.gov (M.L.E.-B.); craig.skinner@ars.usda.gov (C.B.S.); stephanie.patfield@ars.usda.gov (S.A.P.); xiaohua.he@ars.usda.gov (X.H.)

**Keywords:** Keywords: mass spectrometry, Shiga toxins, verotoxins, multiple reaction monitoring method (MRM), STEC, serum, shigatoxigenic and enterohemorrhagic strains of *Escherichia coli*, EHEC, *Enterobacter*

## Abstract

Shiga-like toxins (verotoxins) are responsible for the virulence associated with a variety of foodborne bacterial pathogens. Direct detection of toxins requires a specific and sensitive technique. In this study, we describe a mass spectrometry-based method of analyzing the tryptic decapeptides derived from the non-toxic B subunits. A gene encoding a single protein that yields a set of relevant peptides upon digestion with trypsin was designed. The ^15^N-labeled protein was prepared by growing the expressing bacteria in minimal medium supplemented with ^15^NH_4_Cl. Trypsin digestion of the ^15^N-labeled protein yields a set of ^15^N-labeled peptides for use as internal standards to identify and quantify Shiga or Shiga-like toxins. We determined that this approach can be used to detect, quantify and distinguish among the known Shiga toxins (Stx) and Shiga-like toxins (Stx1 and Stx2) in the low attomole range (per injection) in complex media, including human serum. Furthermore, Stx1a could be detected and distinguished from the newly identified Stx1e in complex media. As new Shiga-like toxins are identified, this approach can be readily modified to detect them. Since intact toxins are digested with trypsin prior to analysis, the handling of intact Shiga toxins is minimized. The analysis can be accomplished within 5 h.

## 1. Introduction

Although the Shiga-like toxins are associated with various bacterial strains, they are actually produced by genes under the control of lambdoid phages that infect the bacterium [1]. This means that when the phage is reproducing lysogenically (reproducing with each of the host’s divisions), toxins are not normally produced or released. If, however, the host’s DNA is damaged or if it is stressed, then the phages reproduce lytically. During lytic replication, the metabolism of the host is diverted to assemble intact phages, express *stx* genes, and produce toxin molecules which all are released when the host cell is ruptured [2]. Bacterial food poisoning by Shiga-like toxin-producing *Escherichia coli (E. coli*) (STEC) is a considerable worldwide health concern [3,4,5].

The phages can produce two types of Shiga-like toxins: Stx1 and Stx2 [6]. Stx1 has a nearly identical amino acid sequence as Shiga toxin (Stx) from *Shigella dysenteriae* [6]. Stx2 differs significantly from Stx1, but is a much more potent toxin *in vivo* [7,8,9]. There are several subtypes of Stx within each type (Stx1: four subtypes; Stx2: seven subtypes), which vary in sequence and toxicity. The multiplicity of Stx types complicates the analysis of toxins.

The interactions of bacterial defense mechanisms coupled with the vagaries of phage replication also complicate the analysis of Shiga-like toxins. The host bacteria may be infected by more than one lambdoid phage (Ф_24B_) and not all those infecting phages are capable of lytic replication and release of toxins [10]. Thus, the presence of a toxin gene does not necessarily mean that it will be expressed. Furthermore, the phages can infect different strains of *E*. *coli* and other species of bacteria [11]. As a result, Shiga-like toxins may be produced by *E*. *coli* strains other than O157:H7 and by other species of bacteria such as *Enterobacter cloacae* and *Citrobacter freundii* [11,12,13]. A recent case of possible foodborne illness was traced back to a Stx-expressing (Stx1e) *Enterobacter cloacae* strain [14]. The production of Shiga-like toxins and retention of the *stx* genes can be transient [15]. All of these constraints complicate the analysis of Shiga-like toxins.

Human serum amyloid P component (HuSAP) provides protection from Stx2 toxins in animal models [16,17]. Mice injected with HuSAP remained healthy after being injected with lethal levels of Stx2 [18]. Transgenic mice expressing HuSAP were similarly protected [18]. This ability to neutralize Stx2 is not found in the serum from other mammals [16,19]. *In vivo* rodent and cell based assays have shown that Stx2 is more a potent toxin that Stx1 [7,8,9]. Detecting Stx1 and Stx2 in human serum may require different approaches, since HuSAP binds Stx2, but not Stx1 [16].

Shiga-like toxins have been detected using a number of different methods. The most common means of detection is to perform PCR on an isolate to determine whether it contains the toxin gene. This approach is limited, since the production of the toxin is regulated by the phage, so the presence of the toxin gene does not guarantee the expression of that toxin. Several antibody-based methods of detecting toxins have been developed, but they are only available for some subtypes [20,21,22,23]. Mass spectrometry has been used to study the structure of the holotoxins [24,25,26,27,28,29,30,31,32,33,34], but has not been used as a method of detection and quantification. Since the toxin is ultimately responsible for the observed symptoms, development of an assay that detects and quantifies all of the Shiga and Shiga-like toxins would be extremely valuable.

Previously, we developed a mass spectrometry based method of detecting Shiga-like toxins in complex matrices [35]. It is based on the well-established multiple reaction monitoring (MRM) method. In the MRM method, samples are digested with proteases to yield sets of characteristic peptides, which are analyzed by a mass spectrometer. Those peptides determined to be suitable are optimized for a MRM method. We use ^15^N-labeled analogs of these peptides as internal standards, since they have identical physico-chemical properties, such as chromatographic retention time and MS fragmentation. In addition, these ^15^N-labeled internal standards can be used to quantitate the amount of analyte peptide in a sample. Since the MRM method does not require the intact protein for analysis [36,37,38], it can be used as a safe and effective means of detecting toxins. The method is extremely sensitive and can detect analyte decapeptides in the attomole (10^−18^ mole) range per injection (~5 femtomole/mL).

In this work we describe how the method can be used to detect Stx2 and Stx1 variants including Stx1a and the recently described Stx1e. We describe how to shorten the time necessary to perform the analysis. We also wish to report the production of a single protein that can be used to easily and cheaply generate the needed ^15^N-lableled tryptic peptide analogs of the unlabeled MRM peptides. In addition, we will show how this method can be used, in conjunction with chaotropes, to detect the presence of Stx1 and Stx2 toxins in complex media, including human serum.

## 2. Results

### 2.1. Generation of the Stx ^15^N-Labeled Internal Standard Protein (^15^N-Stx-ISP)

The ^15^N-labeled analog of a peptide will have the same physico-chemical properties (e.g., chromatographic properties and MS fragmentation) as that of the unlabeled analog. The use of ^15^N-labeled internal standards allows us to identify and quantify only those peaks that have a retention time and MS fragmentation corresponding to that of the internal standards. To simplify the process of generating the multiple internal standards needed to identify all types and subtypes of Shiga toxins detailed in this manuscript, we generated a single gene expressing a protein (Stx internal standard protein (Stx-ISP)) that would yield a useful set of peptides upon tryptic digestion, thus greatly simplifying the process of internal standard generation. These peptides include the analyte decapeptides for Stx2 (YNEDDTFTVK (Stx2a or f), YNENDTFTVK (Stx2b, c, or d), YNEDNTFTVK (Stx2e), and YNGDNTFTVK (Stx2g)); analogous analyte decapeptides for Stx1 (YNDDDTFTVK, YNDDDSFTVK, and YNDDDTFTAK); peptides that can be used to distinguish among the Stx1a and Stx1e subtypes (ELFTNR and ELYTTR); and other peptides that can be used to distinguish among the other Stx1 and Stx2 subtypes. The protein included an *N*-terminal His tag to facilitate purification by immobilized metal affinity chromatography (IMAC). The gene sequence included unique XhoI and NdeI cloning sites to facilitate subsequent subcloning. The sequence was designed [39] to generate a protein that would quantitatively yield the desired peptides. These internal standards are the key to detecting and distinguishing among the known/common Stx2 and Stx1 subtypes.

The BL21 cells were used to express the protein encoded by the *Stx-ISP* gene. They were grown in minimal medium supplemented with ^15^NH_4_Cl to generate, after IMAC, the ^15^N-labeled protein [35,40]. Mass spectrometry-based analysis showed that all of the relevant peptides were observed in the ^15^N samples (*vide infra*). The ^15^N-labeled internal standard was subjected to reduction/alkylation/trypsin digestion and analyzed by mass spectrometry. No signals from unlabeled peptide analogs were observed in samples of ^15^N-labeled material nor were any signals corresponding to the ^15^N-labeled material observed in the unlabeled material. The area ratios indicated that the peptides were at least 99.4% isotopically pure.

### 2.2. MRM Optimization of Stx1 Related Tryptic Peptides

The multiple reaction monitoring method (MRM) is a highly specific and selective method that is routinely used to identify and quantify multiple specific peptides from a complex mixture [35,36,37,38]. This method employs a triple quadrupole mass spectrometer. The first quadrupole is used as a filter to select only those peptides with a mass to charge ratio of interest (*m*/*z*). The second quadrupole is used to fragment those selected peptides using a specified collision energy. The resulting fragment ions pass into the third quadrupole, where they are filtered again according to predetermined *m*/*z*, to select only those daughter ions of interest for detection. The mass spectrometer cycles through each set of *m*/*z* filters (transitions) in as little as 50 milliseconds, so several transitions can be measured in a short time; hence, the “multiple” in multiple reaction monitoring. In order for this method to be effective, peptides of interest must be produced and optimized for collision energy and various other parameters.

To this end, five tryptic peptides (YNDDDTFTVK, YNDDDSFTVK, YNDDDTFTAK, ELFTNR and ELYTTR) were commercially synthesized and obtained in at least 95% purity. The source parameters and the Q2 offset voltage (“collision energy”) were adjusted separately for each peptide to yield optimal fragmentation. The optimized parameters for analyte decapeptides for Stx2 have been previously reported [35]. The analyte decapeptides for Stx1, YNDDDTFTVK, YNDDDSFTVK, and YNDDDTFTAK, were observed to fragment in very similar fashions to those of Stx2 and in all cases the y_8_ fragment ion [41] (containing amino acids DDDTFTVK (*m*/*z* = 940.1; *z* = 1), DDDSFTVK (*m/z* = 926.4; *z* = 1), or DDDTFTAK (*m*/*z* = 891.4; *z* = 1), respectively) produced the most intense signals, so it was used for quantitation. The b_2_ ion (YN (*m*/*z* = 281.1; *z* = 1)) was very intense, but was not used for quantitation due to the small size of the fragment and less specific nature of the YN daughter ion. Similar conditions were optimal for the production of the y_7_ and y_6_ ions (Table S1) from these three decapeptides.

Two other tryptic peptides, ELFTNR and ELYTTR, are produced by the digestion of Stx1a and Stx1e, respectively. The optimal fragments of the ELFTNR peptide were the y_4_ (*m*/*z* = 537.3; *z* = 1), a_2_ (*m*/*z* = 215.1; *z* = 1), and y_2_ (*m*/*z* = 289.2; *z* = 1) ions (containing the amino acids FTNR, EL and TNR, respectively) [41]. For the ELYTTR peptide, the optimal fragments were the y_4_ (*m*/*z* = 540.3; *z* = 1), y_3_ (*m*/*z* = 377.2; *z* = 1), and y_2_ (*m*/*z* = 276.2; *z* = 1) ions (corresponding to amino acids YTTR, TTR, and TR, respectively). The y_4_ ions from these peptides were used to detect and quantitate these peptides in subsequent experiments. These data are summarized in Table S1.

### 2.3. Using Characteristic Stx1 Tryptic Peptides to Distinguish between the Stx1 Subtypes

The analyte decapeptides YNDDDTFTVK, YNDDDSFTVK, and YNDDDTFTAK associated with Stx1 have different precursor ion masses (609.3 (*m*/*z*; *z* = 2), 602.3 (*m*/*z*; *z* = 2), and 595.3 (*m*/*z*; *z* = 2), respectively), which make them readily distinguishable from one another. All three differ from the four analyte decapeptides from Stx2 (616.3, 615.8, 615.8, and 579.8 (*m*/*z*; *z* = 2)), which permits the ready distinction between Stx1 and Stx2. The signals from the y_8_ ions (Table S1) from the tryptic digest of the ^15^N-labeled Stx internal standard protein (^15^N-Stx-ISP) show only a single signal even though all of the other analyte decapeptides are present in those samples (Figure S1). This indicates that the analyte decapeptides can be used to detect the known Stx1 and Stx2 variants.

Distinguishing between Stx1a and Stx1e is complicated by the fact that they possess the same analyte decapeptide (YNDDDTFTVK). Trypsin digestion of Stx1a or Stx1e yields the peptides ELFTNR or ELYTTR, respectively. Stx1a produces the peptide ELFTNR with a precursor ion of 390.2 (*m/z*; *z* = 2) and a product ion of 537.3 (*m*/*z*; *z* = 1; y_4_), while Stx1e produces ELYTTR (precursor ion 391.7 (*m*/*z*; *z* = 2); product ion 540.3 (*m*/*z*; *z* = 1; y_4_)), which can be used to distinguish the two. Analysis of the trypsin digest of the ^15^N-Stx-ISP showed that each peptide had a single signal and that they had different chromatographic retention times (Figure S2). Again, these signals were derived from a sample containing all of the other tryptic peptides. In this way, Stx1 and Stx2 subtypes can be distinguished and Stx1a and Stx1e can be distinguished from one another.

### 2.4. Quantitating Stx1 Toxins and Their Subtypes

The MRM method can be used both to detect the presence of Stx1 toxins and to quantitate the amount of toxin in the sample, by adding a fixed amount of the appropriate ^15^N-labeled internal standard decapeptide to each sample. The UV/Vis absorptions of the three commercially synthesized Stx1 analyte decapeptides were used to calculate the concentration of solutions containing those peptides. Those solutions were mixed with a fixed amount of the tryptic digest of the ^15^N-Stx-ISP to yield samples with 20, 50, 100, 1000, or 10,000 attomoles of the Stx1 analyte decapeptides per injection. The samples were analyzed by mass spectrometry. The area ratios of the y_8_ ions from the three synthetic analyte decapeptides to their corresponding ^15^N-labeled decapeptides were determined. These values were used to prepare calibration curves that permit us to quantitate the amount of Stx1 in a sample (Figure S3).

The peptides ELFTNR and ELYTTR can be used to distinguish between Stx1a and Stx1e, respectively. An analogous procedure was used to prepare calibration curves based on the signal of the y_4_ ions of ELFTNR and ELYTTR peptides to a fixed amount of their corresponding ^15^N-labeled analogs. These values were used to prepare the calibration curves (Figure S4). These results indicated that we could both quantitate the Stx1 present in a sample and distinguish between Stx1a and Stx1e. Both sets of calibration curves were linear with excellent correlation coefficients (*R*^2^ > 0.990).

### 2.5. Shortening the Reduction/Alkylation/Trypsin Time

Our previously described method had an overnight digestion with trypsin. We tested whether our standard reduction/alkylation and subsequent trypsin digestion procedure could be shortened. Samples of bacterial broth supernatants from *E. coli* producing Stx2a, Stx2c, Stx2e, Stx2g, or Stx1a were reduced/alkylated and trypsin digested, in solution, for either the standard period of time (overnight) or a shortened period (2 h). The resulting samples were analyzed and quantitated by mass spectrometry. These results are summarized in Table 1. We determined that shortening the reduction/alkylation period and the trypsin digestion period did not result in a statistically significant reduction in signal (*p* > 0.25). We concluded that shortening the total time to reduce/alkylate and trypsin digest a sample to 3 h resulted in a digestion that was equivalent to and in some cases better than 2 h of reduction/alkylation followed by an overnight digestion.

**Table 1:** Table of the amount of Shiga-like toxins detected after a 2 h or overnight (ON) digestion with trypsin. Values are reported as fmol/injection.

Toxin	2 h	ON
Stx1a	1.3 + 0.2	0.5 + 0.1
Stx1e	0.2 + 0.1	0.2 + 0.1
Stx2g	0.9 + 0.5	0.5 + 0.1
Stx2e	0.11 + 0.02	0.05 + 0.02
Stx2a	4.2 + 0.9	5.0 + 1.7
Stx2c	1.2 + 0.2	1.1 + 0.1

### 2.6. Detection of Stx1a, Stx1e, Stx2a, Stx2c, Stx2g, and Stx2e in Bacterial Broths

**Figure 1:**
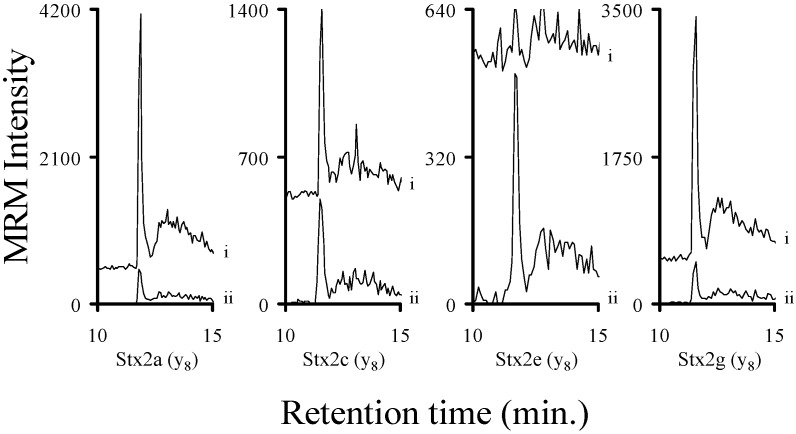
Chromatograms of the MRM signal intensities derived from the y_8_ ions from the analyte decapeptides of Stx2a (YNEDDTFTVK), Stx2c (YNENDTFTVK), Stx2e (YNEDNTFTVK), and Stx2g (YNGDNTFTVK). The samples are derived from sterile filtered TSB broths from Stx2a, Stx2c, Stx2e, or Stx2g producing *Escherichia coli* strains and analyzed by mass spectrometry. The signal from the natural abundance decapeptide (i) is from the toxin and is not normalized. The corresponding signal from the ^15^N-labeled decapeptide is from the added internal standard (ii) and is included to show the retention time of the analyte decapeptide and is normalized to 500 to allow the signal from the analyte decapeptide to be viewed on the same plot.

**Figure 2:**
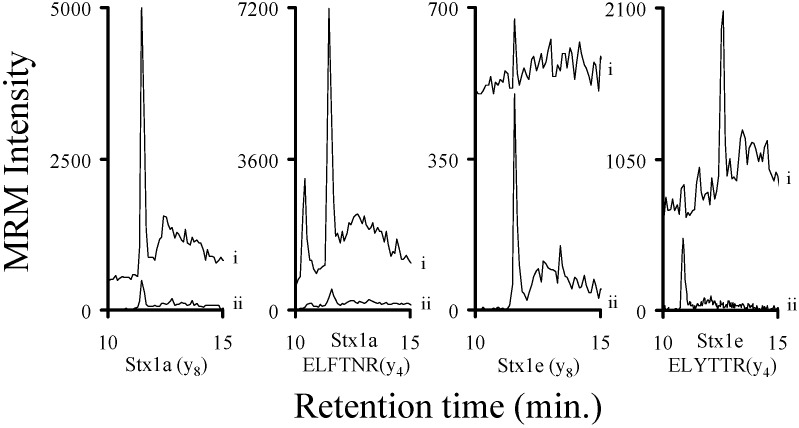
Chromatograms of the MRM signal intensities derived from the y_8_ ions from the analyte decapeptide (YNDDDTFTVK; Stx1a and Stx1e) or the y_4_ ions from ELFTNR (Stx1a) or ELYTTR (Stx1e). The samples are derived from the TSB broth of a Stx1a producing *Escherichia coli* or the LB broth from a Stx1e producing *Enterobacter cloacae* strain and analyzed by mass spectrometry. The signal from the natural abundance peptide (i) is from the toxin and is not normalized. The signal from the corresponding ^15^N-labeled peptide is from the added internal standard (ii) and is normalized to an intensity of 500 to allow the signal from the peptide to be viewed on the same plot.

Bacterial cultures known to produce Stx1a, Stx1e, Stx2a, Stx2c, Stx2e, and Stx2g were grown in a manner to facilitate the production of these toxins. The filter sterilized samples were processed (reduction/alkylation/trypsin digestion) and then analyzed by mass spectrometry. The chromatograms of the y_8_ ions (Table S1) of the analyte decapeptides from the Stx2 containing samples are shown in Figure 1. The chromatograms of the y_8_ ion (Table S1) from the analyte decapeptide from Stx1a and Stx1e are shown in Figure 2. In addition, the signals from y_4_ ions (Table S1) of the ELFTNR and ELYTTR peptides (Stx1a and Stx1e, respectively) are included in Figure 2. All of the Stx2 subtypes were readily detected in the TSB medium. The Stx1a and Stx1e samples were readily detectable in the LB medium. In addition, the signals from the ELFTNR and ELYTTR peptides were used to distinguish between Stx1a and Stx1e, respectively. The amount of toxin in the samples varied from approximately 2.5 fmol/mL (Stx1e) to 530 fmol/mL (Stx1a). This indicates that this method can be used to detect Shiga-like toxins in complex bacterial media.

### 2.7. Assessing the Background in Human Serum

**Figure 3:**
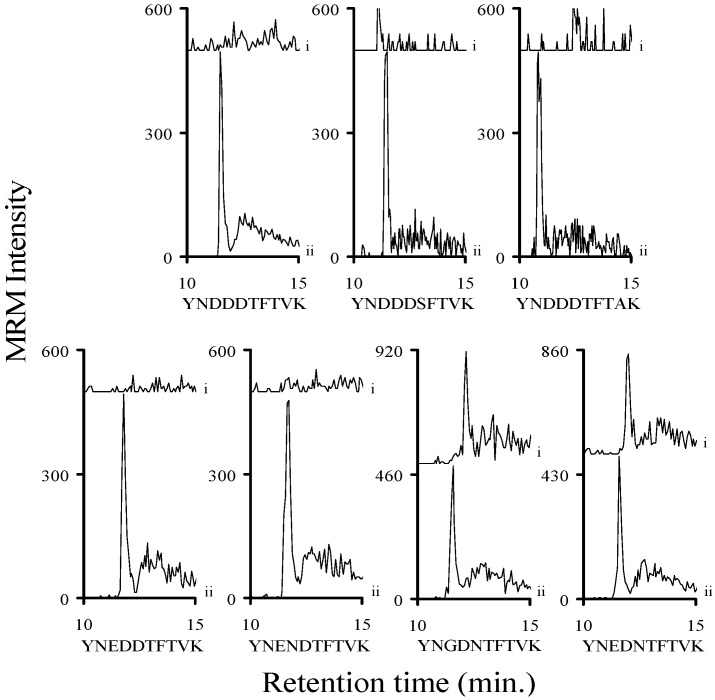
Chromatograms of the MRM signal intensities derived from the y_8_ ions of the analyte decapeptides from tryptic digests of human serum showing no matrix interference with the y_8_ ion of the analyte decapeptides from Stx1 and Stx2 subtypes. The signals corresponding to the y_8_ ion of the analyte decapeptides from Stx1 (YNDDDTFTVK, YNDDDSFTVK, or YNDDDTFTAK); or Stx2a and Stx2f (YNEDDTFTVK); Stx2b, Stx2c, or Stx2d, (YNENDTFTVK); Stx2g (YNGDDTFTVK) or Stx2e (YNEDNTFTVK) are shown in each graph (i). Each sample was spiked with the corresponding ^15^N-labeled internal standard (ii). The signals for the y_8_ ions from the corresponding ^15^N-labeled internal standards (ii) are normalized to 500 to allow the signal from the analyte decapeptide to be viewed on the same plot. The other signals are not normalized.

The trypsin digestion of human serum will produce a large number of diverse tryptic peptides, so it is possible that some of those molecules may interfere with our analysis. Samples of human serum were digested with trypsin and then spiked with the trypsin digested ^15^N-Stx-ISP. These spiked samples were then analyzed by mass spectrometry. The resulting chromatograms were examined to determine if there was a molecule present in the trypsin digested human serum that would interfere with this analysis. None of these chromatograms showed a signal with the same precursor/product ion and the same chromatographic retention time as the corresponding ^15^N-labeled internal standard. These chromatograms of the y_8_ ions (Table S1) from the Stx1 and Stx2 analyte decapeptides are shown in Figure 3. The chromatograms from either the y_3_ or y_4_ ions (Table S1) from the IEFSK, VEYTK, ELFTNR, EYWTSR, EYWTNR, or ELYTTR peptides are shown in Figure S5. When we previously examined hamster, sheep and mouse plasma, we saw no interference from molecules present in these matrices [35]. It was determined that there were no molecules present in the serum sample that would interfere with the analysis.

### 2.8. Determining a LOD for Relevant Stx1 Peptides in Buffer and Serum

**Figure 4:**
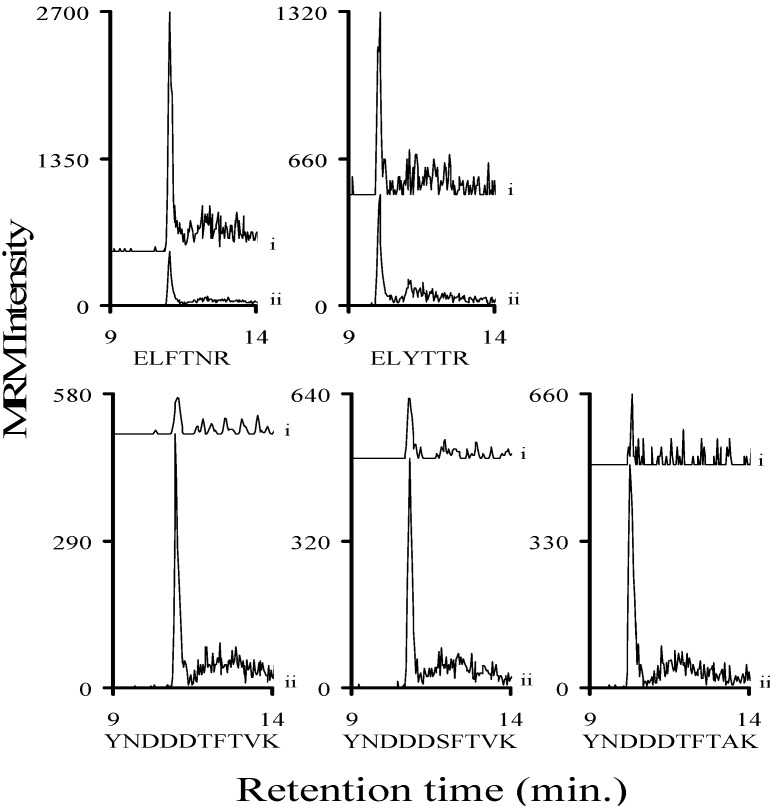
Signal intensity of a 50-attomole injection of each of the three synthetic Stx1 analyte decapeptides (YNDDDTFTVK, YNDDDSFTVK, and YNDDDTFTAK), the ELFTNR, or the ELYTTR peptides in buffer (8% acetonitrile, 0.01% BOG, 25 mM ABC pH 8). Graphs show the MRM signal intensity derived from the y_8_ ion of the analyte decapeptides or the y_4_ ions from the other peptides from a (i) 50-attomole injection. The corresponding ^15^N-labeled analog peptides are normalized to a signal intensity of 500 (ii) to allow the signal from the analyte decapeptide to be viewed on the same plot.

**Figure 5:**
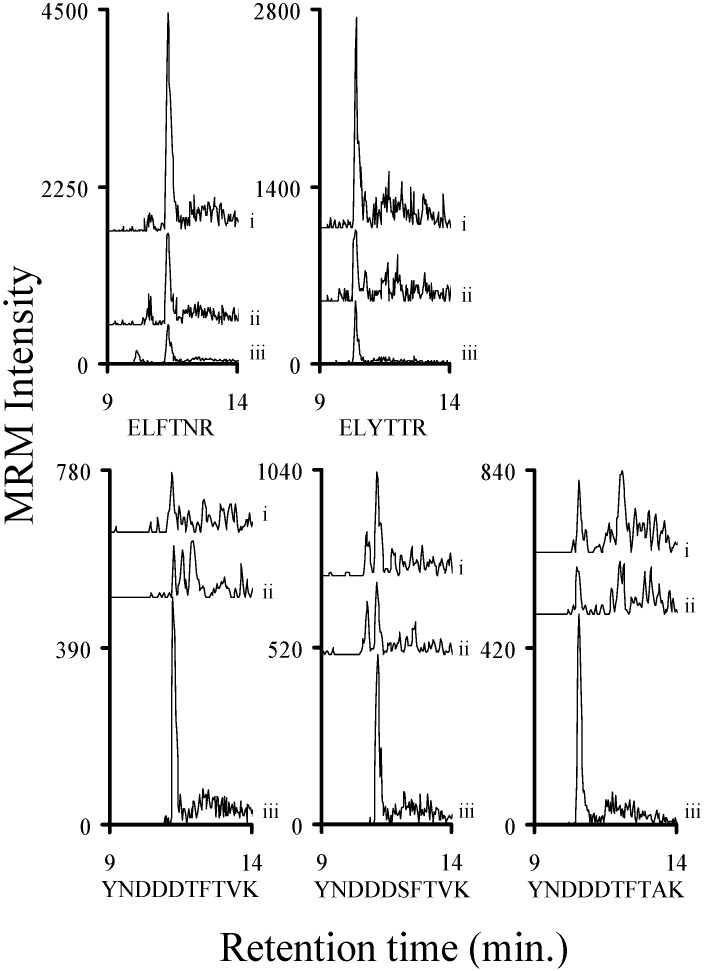
Signal intensity of a 50- or 100-attomole injection of each of the three synthetic Stx1 analyte decapeptides (YNDDDTFTVK, YNDDDSFTVK, and YNDDDTFTAK), the ELFTNR or the ELYTTR peptides in human serum. Graphs show the MRM signal intensity derived from the y_8_ ion of the analyte decapeptides or the y_4_ ions for the other peptides from a (i) 50- or (ii) 100-attomole injection. The corresponding ^15^N-labeled analog peptides are normalized to a signal intensity of 500 (ii) to allow the signal from the analyte decapeptide to be viewed on the same plot.

After we established that the peptides present in trypsin digested human serum do not interfere with our analysis, we determined the limit of detection (LOD) of the Stx1 peptides in buffer and human serum. The LOD values represent signals that were at least 3× greater than the noise. Known concentrations of the relevant commercially synthesized Stx1 peptides (YNDDDTFTVK, YNDDDSFTVK, YNDDDTFTAK, ELFTNR, and ELYTTR) were diluted into buffer (25 mM ammonium bicarbonate, pH 8.0; 0.01% β-octylglucopyranoside; and 8% acetonitrile) or trypsin digested human serum along with the analogous ^15^N-labeled peptides. After these samples were analyzed by mass spectrometry we determined that the limit of detection for the analyte decapeptides was less than 50 attomoles/injection in buffer and in trypsinized human serum (Figure 4 and Figure 5). The LOD for the ELFTNR and ELYTTR peptides was noticeably lower than the analyte decapeptides in both buffer and serum. The chromatograms of the y_8_ ions of the analyte decapeptides and the y_4_ ions of the ELFTNR and ELYTTR peptides (Table S1) are shown in Figure 4 and Figure 5. Shiga-like toxins have five B subunits per toxin molecule, so a LOD of less than 50 attomoles/injection for a peptide corresponds to a LOD for the toxin of less than 10 attomoles/injection. A three-fold loss occurs when a toxin is digested with trypsin (*vide infra*). Since each injection consists of six microliters and the molecular weight of Shiga toxins is about 70,000, the LOD of the toxin corresponds to approximately 40 pg/mL, when the loss due to digestion in serum is taken into account.

Serum samples were filtered and then spiked with toxins to determine if the interfering protein or proteins could be removed by filtration. Serum samples were either filtered through a 300,000 Da (300 K) or 100,000 Da (100 K) molecular weight cutoff (MWCO) filter or unfiltered before being spiked with Stx1a or Stx2a. Subsequent mass spectrometry-based analysis showed that Stx1a was readily detectable in all of the samples, whether filtered or unfiltered. There was a notable loss when the Stx1a was spiked into serum and then filtered. In contrast, Stx2a was readily detectable only in the serum samples that were filtered prior to spiking (Table 2). This indicates that the serum matrix effect is due to the binding of Stx1 or Stx2 to a large molecule, which then prevents the tryptic digestion of the intact toxin, and that this effect is more pronounced with Stx2a.

**Figure 6:**
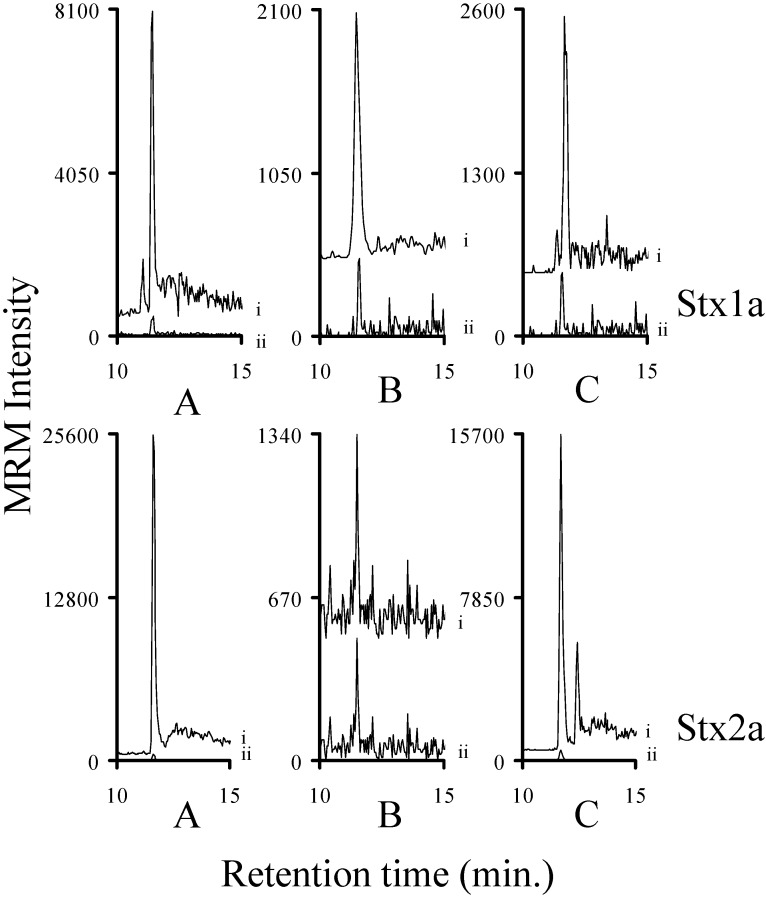
The intensity of the MRM signals derived from the y_8_ ion of the analyte decapeptide of Stx1a or Stx2a (i) in sterile filtered bacterial medium (**A**); human serum (**B**); and human serum denatured with guanidinium chloride (**C**). The corresponding ^15^N-labeled internal standard is normalized to 500 (ii) to allow the signal from the analyte decapeptide to be viewed on the same plot. The amount of Stx1a in each injection (*n* = 2) was calculated to be (left (l) to right (r)) 5.8 ± 0.4 fmol, 3 ± 1 fmol, and 1.7 ± 0.8 fmol, respectively. The amount of Stx2a in each injection (*n* = 2) was calculated to be (l to r) 29 ± 0.4 fmol, 0.08 ± 0.05 fmol, and 10 ± 0.6 fmol, respectively.

**Figure 7:**
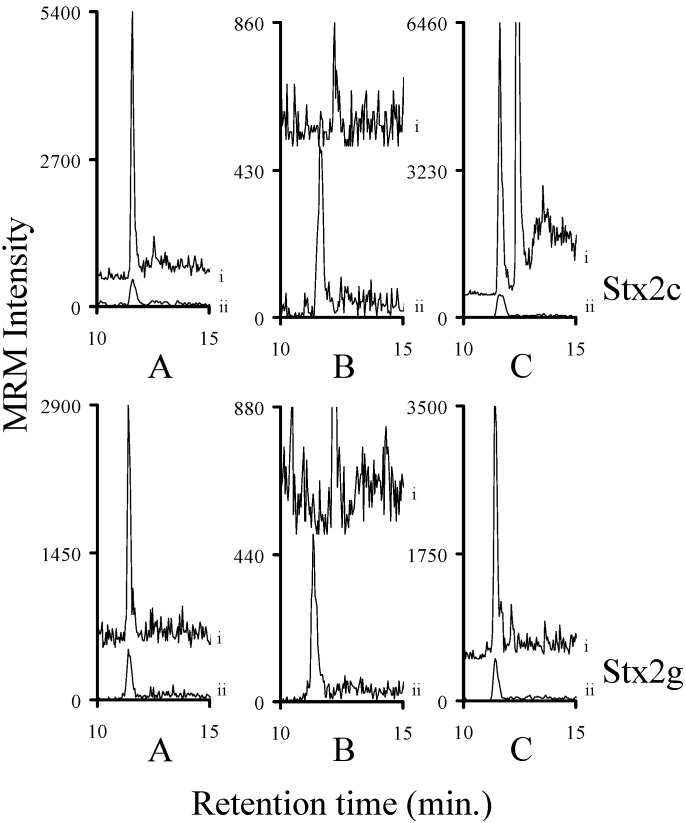
The intensity of the MRM signals derived from the y_8_ ion of the analyte decapeptide of Stx2c or Stx2g (i) in sterile filtered bacterial medium (**A**); human serum (**B**); and human serum denatured with guanidinium chloride (**C**). The corresponding ^15^N-labeled internal standard is normalized to 500 (ii) to allow the signal from the analyte decapeptide to be viewed on the same plot. The amount of Stx2c in each injection (*n* = 2) was calculated to be (left (l) to right (r)) 34 ± 3 fmol, 0.02 ± 0.01 fmol, and 28 ± 7 fmol, respectively. The corresponding amount of Stx2g in each injection (*n* = 2) was calculated to be (l to r) 4.1 ± 0.3 fmol, <0.01 fmol, and 3 ± 0.2 fmol, respectively.

Guanidinium chloride (GuCl; 6 M) was used to disrupt the interaction between the molecule or molecules (presumably HuSAP) and Stx2. Human serum samples were spiked with varying amounts of Stx1a, Stx2a, Stx2c or Stx2g. The samples were dissolved in GuCl where they were reduced/alkylated and then methanol precipitated. The resulting pellet was digested with trypsin and then analyzed by mass spectrometry. Using GuCl to disrupt the interaction allows us to detect previously inaccessible amounts of Stx2 in human serum (Figure 6 and Figure 7). The ratio of the amount of toxin detected in the broth spike to the amount of toxin detected in the broth spike in serum was calculated for four GuCl treated samples: Stx1a (0.3 ± 0.1; *n* = 4), Stx2a (0.3 ± 0.04; *n* = 4), Stx2c (0.8 ± 0.2; *n* = 4), and Stx2g (0.7 ± 0.06; *n* = 4). This procedure substantially improved the sensitivity of detection of Stx2a, Stx2c, and Stx2g samples. However, approximately 20%–70% of the Stx toxin in the sample is lost during this process. Since the samples can be concentrated, it is possible that this loss may be overcome by using a larger sample.

**Table 2:** Calculated proportion of the spiked toxin (Stx1a or Stx2a) detected in filtered human serum relative to that detected in unfiltered spiked human serum (HS). Identical amounts of Stx1a or Stx2a were spiked into human serum that was unfiltered or filtered through a 100,000 Da (100 K) or 300,000 Da (300 K) MWCO filter. The resulting samples were reduced/alkylated/trypsin digested and then analyzed by mass spectrometry.

Sample Description	Proportion
Stx1a + HS	1.00
Filtered HS (100 K) + Stx1a	0.99
Stx1a + HS, Filtered (100 K)	0.13
Filtered HS (300 K) + Stx1a	0.59
Stx1a + HS, Filtered (300 K)	0.15
Stx2a + HS	1
Filtered HS (100 K) + Stx2a	326
Stx2a + HS, Filtered (100 K)	14
Filtered HS (300 K) + Stx2a	115
Stx2a + HS, Filtered (300 K)	5

## 3. Discussion

### 3.1. The Stx ^15^N-Labeled Internal Standard Protein

The Stx ^15^N-labeled internal standard protein (^15^N-Stx-ISP) is composed of analyte decapeptides and other peptides that are produced by the trypsin digestion of B subunits from the known Stx1 and Stx2 toxins. The B subunits bind the toxin to the gangliosides present on the external cell membrane, but are not toxic by themselves. The ^15^N-Stx-ISP combines the unique peptides derived from the three B subunits of Stx1 subtypes and four B subunits of Stx2 subtypes as well as other peptides into a single protein. To facilitate purification by immobilized metal affinity chromatography (IMAC), it has an *N*-terminal His-tag. The host BL21 cells can be grown in minimal medium, supplemented with ^15^NH_4_Cl, to yield the corresponding ^15^N-labeled His-tagged protein. In this way, the needed ^15^N-labeled internal standard peptides can be readily and cheaply produced using standard molecular biology techniques. As Stx variants emerge, the gene can be readily modified to incorporate the needed peptides.

The internal standards were derived from cloned proteins, so we needed to determine if the other peptides produced by the trypsin digestion of these proteins would interfere with the analysis. Samples of four purified ^15^N-labeled His-tagged B subunit proteins from Stx1a, Stx2a, Stx2c, Stx2e, and Stx2g were analyzed by our mass spectrometry-based method. We determined that none of the tryptic peptides derived from the ^15^N-Stx-ISP interfered with the analysis of the Stx, Stx1, or Stx2 toxins. Additionally, we injected approximately 100 fmol of the tryptic peptides from each of the five ^15^N-labeled internal standards and analyzed them by mass spectrometry. The resulting chromatograms were examined and we saw no signal above noise for the corresponding natural abundance (^14^N) peptide (data not shown), which indicated that the ^15^N-labeled internal standards were of very high isotopic purity. In summary, the ^15^N-Stx-ISP is of high isotopic purity and, upon trypsin digestion, yields no peptides that would interfere with this analysis.

### 3.2. Assessing the Background Interference with the Analyte Peptides

The trypsin digestion of a toxin resulted in a large mixture of non-analyte tryptic peptides, so it was necessary to determine if these other peptides would interfere with the analysis. Six toxins (Stx1a, Stx1e, Stx2a, Stx2c, Stx2e, and Stx2g) were digested with trypsin and analyzed by mass spectrometry. This analysis indicated that the other peptides derived from the tryptic digestion of the Stx1 toxin did not interfere with the detection of the Stx2 subtypes. The converse was also true.

It was necessary to determine whether any molecules present in the media (Tryptic soy broth or Luria broth) where STEC are grown to induce toxin production or human serum might interfere with this analysis. An interfering molecule would have to have an identical chromatographic retention time and identical precursor and product ions. The samples were spiked with the appropriate ^15^N-labeled internal standards. Upon examination of the resulting chromatograms, no interfering molecules were found.

### 3.3. Calibration Curves for the Quantification of Stx1 Toxins in a Sample

The amount of synthetic analyte decapeptide provided by the vendor was determined by measuring its absorbance at 280 nm, when appropriate, or by using the analysis provided by the vendor. The ^15^N internal standards were obtained from the trypsin digest of the ^15^N-Stx-ISP. A set of solutions containing a fixed amount of the ^15^N-labeled internal standard and varying amounts of the corresponding synthetic peptide were prepared for each of the three Stx1 analyte decapeptides and ELFTNR and ELYTTR. These solution sets were analyzed by mass spectrometry. The area ratio of the signal from the y_8_ ion of the synthetic peptide to the y_8_ ion of the analogous ^15^N-labeled internal standard was calculated (*n* = 4) for each solution. These data were used to prepare a calibration curve relating the area ratio of the signal from the y_8_ ion from a known amount of synthetic peptide to the y_8_ ion from a fixed amount of the corresponding isotopically labeled internal standard. Analogous curves were prepared using the y_4_ ions from the ELFTNR and ELYTTR peptides. The curves (Figures S3 and S4) were determined to be linear over a >100-fold range with excellent correlation coefficients (>0.99).

### 3.4. Quantifying Shiga-Like Toxins in Bacterial Media

Clinical isolates of STEC producing Stx1a, Stx1e, Stx2a, Stx2c, Stx2e, or Stx2g were grown in LB or TSB and induced to produce Shiga-like toxin by the addition of the antibiotic mitomycin C [42]. An aliquot of the filter sterilized supernatants from each of these samples was digested with trypsin and a fixed amount of the appropriate trypsin digested ^15^N-labled internal standard was added. The six samples were analyzed by mass spectrometry and the area ratio of the signal from the y_8_ ion of the analyte to the corresponding ^15^N-labeled decapeptide was calculated. The previously described calibration curves were used to quantify the amount of toxin present in the ten previously described samples. These data are listed in the legends of Figure 1 and Figure 2. We were able to detect and quantify all of the Stx2 toxin subtypes in the attomole range (20 (Stx1e) to 960 (Stx2g) attomoles)) (Figure 1). We were also able to detect Stx1 at similarly low levels (Figure 2). In all cases, we were able to detect and quantify toxins in the attomole range.

### 3.5. Quantifying Shiga Like Toxins in Human Serum

Bacterial medium containing Stx2a or Stx1a was spiked into human serum and analyzed by mass spectrometry. We were able to detect and quantify the amount of Stx1a and Stx2a in the samples. A component of the serum (presumably HuSAP) significantly impeded the trypsin digestion of Stx2a. This could be overcome by using GuCl to denature the proteins in the sample and prevent their refolding by reducing and alkylating them. Methanol precipitation removed the GuCl and trypsin digestion released the analyte decapeptides. This approach allows a sample to be concentrated prior to analysis, which permits a larger volume sample to be analyzed with a resulting increase in sensitivity. In addition, the use of filtration to retain the Stx2/HuSAP complex for subsequent analysis using GuCl may further increase the sensitivity of this method.

## 4. Experimental Procedures

### 4.1. Generation of Shiga-Like Toxin Samples

The strains of Shiga-like toxin producers used in this study have been previously described [35]. For each strain, a single colony was used to inoculate a 20 mL culture of tryptic soy broth or Luria Broth (TSB or LB; Fisher Scientific, Pittsburgh, PA, USA) and grown overnight at (37 °C and 250 rpm). The next day 10 mL of the overnight culture was used to inoculate 500 mL of TSB or LB supplemented with 50 ng/mL of mitomycin C (Sigma-Aldrich, Milwaukee, WI, USA) and grown for 24 h (37 °C, 250 rpm). For the Stx2 producing strains, the resulting culture was centrifuged for 15 min at 5000× *g*. For the Stx1 producing strains the culture was diluted 1:1 with Phosphate B-PER (Thermo Scientific, Waltham, MA, USA) and then centrifuged for 15 min at 5000× *g*. The resulting supernatants were sterile filtered and used for mass spectrometry-based analysis (*vide infra*) [43].

### 4.2. Preparation of Serum Samples

The human serum samples were obtained from an FDA licensed commercial donor center and processed in an FDA registered facility (Innovative Research, Inc., Novi, MI, USA). They were reduced/alkylated/trypsin cleaved (*vide infra*).

Human serum samples were processed through 100,000 Da or 300,000 Da MWCO centrifugal filters before reduction/alkylation/trypsin digestion. One hundred microliters of serum was placed in the filter and centrifuged for 20 min at 14,000× *g*. Ten microliters of serum filtrate was spiked with 10 µL of sterile-filtered, toxin-containing supernatant in broth, then reduced/alkylated/trypsin digested and processed for mass spectrometry-based analysis. Alternately, 50 µL of serum was combined with 50 µL of toxin-containing supernatant, and then filtered through 100,000 Da or 300,000 Da MWCO filters for 20 min at 14,000× *g*. Twenty microliters of the filtrate was then reduced/alkylated/trypsin digested and subsequently analyzed by mass spectrometry. Filtrates were stored at −20 °C until analysis.

Forty microliters of toxin-containing supernatant, alone or with 10 µL of serum, was diluted with 150 µL of 8 M guanidinium chloride and mixed. Samples were reduced (adding 5 µL 1 M DTT; 30 min; 37 °C) and alkylated (37 µL of a 500 mM iodoacetamide; 30 min; room temperature in the dark), followed by quenching with DTT (adding 6 µL 1 M DTT; room temperature). These samples were methanol precipitated by the addition of ice-cold methanol to give a final concentration of 85% methanol and allowed to precipitate for 1 h at −20 °C. Samples were centrifuged (10 min; 20,000× *g*; 0 °C). The methanol supernatant was removed and the pellets were washed with methanol and centrifuged (10 min; 20,000× *g*; 0 °C). The methanol supernatant was removed and pellets were air dried briefly and then brought up in 90 µL of buffer A (25 mM ammonium bicarbonate pH 8.0, 8% acetonitrile, 0.01% β-octylglucopyranoside). Ten microliters of a trypsin solution (20 µg/mL) was added to each sample and they were incubated for 2 h at 37 °C. Samples were then processed for mass spectrometry-based analysis. Filtrates were stored at −20 °C until analysis.

### 4.3. Reduction, Alkylation, and Tryptic Cleavage of Shiga-Like Toxins

The sample preparation has been described previously [35]. The samples consisted of one of the following: 20 µL of sterile filtered bacterial supernatant, or 10 µL of serum plus 10 µL of undiluted or diluted bacterial supernatant.

The reduction/alkylation/trypsin cleavage has been described previously [35]. All solutions were freshly prepared prior to use. Ten microliters of 15 mM dithiothreitol (DTT) in buffer A (25 mM ammonium bicarbonate, pH 8.0; 0.01% β-octylglucopyranoside; and 8% acetonitrile) was added to the sample solution and incubated for 0.5 or 1 h at 37 °C and then cooled to room temperature. Forty microliters of 22 mM iodoacetamide (IA) solution in buffer A was added to the DTT solution and allowed to stand in the dark at room temperature for 0.5 or 1 h. Addition of 20 μL of a 22 mM solution of DTT in buffer A quenched the excess IA. The reduced and alkylated proteins were digested with trypsin solution (10 μL of a solution containing 100 μg of trypsin/mL of water) at 37 °C for either 2 or 16 h. After digestion, the samples were filtered through a 10,000 Da MWCO filter (12 min; 14,000× *g*) followed by a 3000 Da MWCO filter (20 min; 14,000× *g*) and stored at −20 °C for eventual analysis.

### 4.4. Mass Spectrometry

The instrument response was optimized by a previously described method [44]. The qualitative mass spectrometry was performed using a Thermo Scientific (Thermo Fisher Scientific, Waltham, MA, USA) model Orbitrap Elite instrument equipped with a nanoelectrospray source. A 4000 Q-Trap (Sciex, Dublin, CA, USA) mass spectrometer equipped with a nanoelectrospray source was used for quantification. This mass spectrometer was operated in multiple reaction monitoring (MRM) mode, alternating between detection of the nine peptides and the corresponding ^15^N-labeled internal standards. The mass settings for the peptides are summarized in Table S1. The retention times of the peptides are listed in Table S2. Quantification was done with the IntelliQuan quantification algorithm using Analyst 1.5 software (Sciex, Dublin, CA, USA).

### 4.5. Nanospray LC-MS/MS

A 4000 Q-Trap mass spectrometer (Sciex, Dublin, CA, USA) equipped with a nanoelectrospray source was used to perform nanospray liquid chromatography and tandem mass spectroscopy (LC-MS/MS). An aliquot (6 μL) of each digest was loaded onto a C-18 trap cartridge (Acclaim PepMap100, 5 μm, 100 Å, 300 μm (inside diameter) × 5 mm (Dionex, Sunnyvale, CA, USA)). Salts were washed from the cartridge with an acetic acid/acetonitrile/heptafluorobutyric acid/water solution (0.5/1/0.02/99). The now salt-free bound peptides were eluted onto a reversed-phase column (Vydac (Hesperia, CA, USA) 238EV5.07515, 75 μm × 150 mm). The solvents were delivered with a Tempo nanoflow LC system (Dionex, Dublin, CA, USA) with an autosampler, a column switching device, and a nanoflow solvent delivery system. Samples were eluted from the column with a binary gradient (A, 0.5% acetic acid in water, and B, 80% acetonitrile with 0.5% acetic acid). The flow rate was 250 nL/min with a 16 min linear gradient starting with 5% B and ending with 100% B. Elution with 100% B was conducted for 7 min followed by a return to 5% B over 4 min. The eluted samples were sprayed with a non-coated spray tip (FS360-20-10-N-20-C12, New Objective Inc., Woburn, MA, USA) onto the Nanospray II source (Sciex, Dublin, CA, USA).

### 4.6. Preparation of the Gene Containing the Internal Standards [45]

A gene was designed to yield a protein that would liberate the peptides used in this manuscript upon digestion with trypsin (YNDDDTFTVK, YNDDDSFTVK, YNDDDTFTAK, YNGDNTFTVK, YNEDNTFTVK, YNENDTFTVK, YNEDDTFTVK, ELFTNR, EYWTNR, ELYTTR, VEYTK, and IEFSK) and others. The codons for the amino acids were changed from the native sequence to minimize repeat regions and to maintain a G/C and A/T content that was consistent with the *E. coli* host that would overexpress the desired protein. The gene was prepared by Biomatik (Wilmington, DE, USA) and subsequently was excised from the pBlueScript II SK(+) (kan) plasmid and cloned into a pET 15b vector (BLA selection) to yield a construct with an *N*-terminal His tag to facilitate purification. The gene composition was verified by sequencing. The pET15b plasmid was cloned into BL21 cells for overexpression of the encoded protein.

The gene sequence is as follows:

ATGGGCAGCAGCCATCATCATCATCATCACAGCAGCGGCCTGGTGCCGCGCGGCAGCCATATGAGAGCGGATTGTGCCAAGTACAATGACGACGACACCTTTACTGTCAAGATCAAGTATAACGGCGATAATACCTTTACTGTCAAGGCGGACTGTGCCGTCGGCAAGACTCCTGATTGTGTCACTGGTAAGTATAACGATGATGACTCCTTCACCGTGAAGTATAACGAGGATAATACGTTCACCGTTAAGGCCCGTGAGTTGTTTACCAACCGCTACAATGACGACGATACCTTTACTGCGAAGGGCCGCGAGTACTGGACCTCTCGCTATAACGAGAACGATACGTTCACAGTTAAGGCCCGCGAGTACTGGACCAACCGCTACAATGAGGACGATACCTTCACGGTCAAGTCCCGCGAATTATATACTACCCGCGTGGAGTATACTAAGATTGAGTTTTCCAAGAATGGCGAGGGCTTCTCAGAGGTGATTTTTCGCACTAACGCCTGCCACAATGGCGGTGGCTTTAGCGAGGTTATCTTTAGGACTACGGCCTGTCATAACGGAGGCGGTTTCTCTGAGGTCATTTTCCGTTCCTCGACCTGTGAGTCCGGCTCCGGCTTTGCTGAGGTGCAGTTTAACAACGACTAGTGACTCGAG

### 4.7. Peptides, Internal Standards, Purified Proteins, and Calibration Curves

The natural abundance (^14^N) peptides YNDDDTFTVK, YNDDDSFTVK, YNDDDTFTAK, ELFTNR, and ELYTTR were obtained from Elim Biopharmaceuticals (Hayward, CA, USA) or Peptide 2.0 (Chantilly, VA, USA). The structures (Figure S6) were confirmed by mass spectrometry and each was >95% pure. The concentration of the peptide was calculated by using the observed absorbance and the calculated extinction coefficient (ExPASy; [39]) or the manufacturer’s specifications. A solution of 500 or 1000 picomoles/microliter in acetonitrile/water (1:1) buffer was prepared for each peptide. Dilutions of these solutions combined with a fixed aliquot of the trypsin digest of the Stx ^15^N-labeled internal standard protein (^15^N-Stx-ISP) were used to prepare the calibration curves.

The ^15^N-labeled internal standard-containing protein was prepared by growing the clone in minimal medium supplemented with ^15^NH_4_Cl (99.7% ^15^N; Cambridge Isotope, Andover, MA, USA) and purified using standard molecular biology techniques [35]. The purity of the ^15^N-labeled internal standard protein was greater than 90% by Coomassie stained SDS-PAGE gel.

## 5. Conclusions

**Figure 8:**
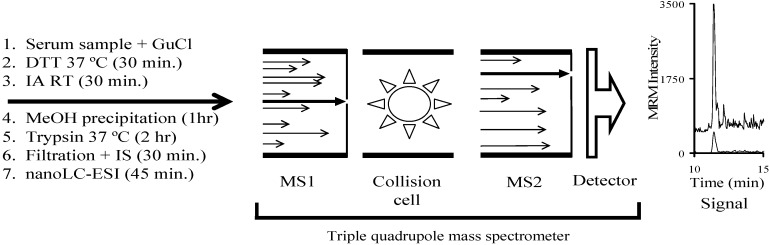
Scheme showing the process of analyzing a human serum sample by mass spectrometry. The serum sample is diluted with the chaotrope guanidinium chloride (GuCl; 6 M) and the sample is then reduced/alkylated with dithiothreitol (DTT) and iodoacetamide (IA), respectively. The sample is methanol precipitated and then brought up in 90 microliters of buffer (8% acetonitrile, 0.01% BOG, 25 mM ABC pH 8) and then digested with trypsin. The trypsin digest is filtered sequentially through a 10,000 and a 5000 Da MWCO filter. The internal standard (IS) is added and the sample is run on the instrument. The appropriate ^15^N-labeled internal standard is used to identify the peak corresponding to analogous unlabeled peptide based on their identical physico-chemical properties (chromatographic retention time and MS fragmentation). The area ratio of the unlabeled peptide to that of the added internal standard may be calculated and used to quantitate the amount of Stx present in the sample. The signal shown is from a human serum sample spiked with Stx2g.

We have developed a mass spectrometry-based method of detecting Stx1 and Stx2 in bacterial media and human serum. We used a synthetic gene to produce a single protein (^15^N-Stx-ISP) to conveniently and cheaply generate the needed ^15^N-labeled internal standards. As new Stx variants emerge they can be added to the gene. This method can be used to distinguish among the known Stx2 and Stx1 subtypes in complex media. When chaotropes are employed, it can be used to detect both Stx1 and Stx2 in human serum. The limit of detection in serum is approximately 5 fmol/mL. The entire procedure can be accomplished in approximately 5 h (Figure 8).

## Supplementary Materials

The following are available online at www.mdpi.com/2072-6651/7/12/4875/s1.

## References

[1] Tyler J.S., Livny J., Friedman D.I., Waldor M.K., Friedman D.I., Adhya S.L. (2005). Lambdoid phages and Shiga toxin. Phages: Their Role in Pathogenesis and Biotechnology.

[2] Friedman D.I., Court D.L. (2001). Bacteriophage lambda: Alive and well and still doing its thing. Curr. Opin. Microbiol..

[3] Cantey J.R. (1985). Shiga toxin—An expanding role in the pathogenesis of infectious diseases. J. Infect. Dis..

[4] Eaton K.A., Friedman D.I., Francis G.J., Tyler J.S., Young V.B., Haeger J., Abu-Ali G., Whittam T.S. (2008). Pathogenesis of renal disease due to enterohemorrhagic *Escherichia coli* in germ-free mice. Infect. Immun..

[5] Tarr P.I., Gordon C.A., Chandler W.L. (2005). Shiga-toxin-producing *Escherichia coli* and haemolytic uraemic syndrome. Lancet.

[6] O’Brien A.D., Tesh V.L., Donohue-Rolfe A., Jackson M.P., Olsnes S., Sandvig K., Lindberg A.A., Keusch G.T. (1992). Shiga toxin: Biochemistry, genetics, mode of action, and role in pathogenesis. Curr. Top. Microbiol. Immunol..

[7] Fuller C.A., Pellino C.A., Flagler M.J., Strasser J.E., Weiss A.A. (2011). Shiga toxin subtypes display dramatic differences in potency. Infect. Immun..

[8] Lindgren S.W., Samuel J.E., Schmitt C.K., O’Brien A.D. (1994). The specific activities of Shiga-like toxin type ii (SLT-II) and SLT-II-related toxins of enterohemorrhagic *Escherichia coli* differ when measured by Vero cell cytotoxicity but not by mouse lethality. Infect. Immun..

[9] Tesh V.L., Burris J.A., Owens J.W., Gordon V.M., Wadolkowski E.A., O’Brien A.D., Samuel J.E. (1993). Comparison of the relative toxicities of Shiga-like toxins type I and type II for mice. Infect. Immun..

[10] Smith D.L., Rooks D.J., Fogg P.C., Darby A.C., Thomson N.R., McCarthy A.J., Allison H.E. (2012). Comparative genomics of Shiga toxin encoding bacteriophages. BMC genomics.

[11] James C.E., Stanley K.N., Allison H.E., Flint H.J., Stewart C.S., Sharp R.J., Saunders J.R., McCarthy A.J. (2001). Lytic and lysogenic infection of diverse *Escherichia coli* and *Shigella* strains with a verocytotoxigenic bacteriophage. Appl. Environ. Microbiol..

[12] Paton A.W., Paton J.C. (1996). *Enterobacter cloacae* producing a Shiga-like toxin II-related cytotoxin associated with a case of hemolytic-uremic syndrome. J. Clin. Microbiol..

[13] Schmidt H., Montag M., Bockemuhl J., Heesemann J., Karch H. (1993). Shiga-like toxin II-related cytotoxins in *Citrobacter freundii* strains from humans and beef samples. Infect. Immun..

[14] Probert W.S., McQuaid C., Schrader K. (2014). Isolation and identification of an *Enterobacter cloacae* strain producing a novel subtype of Shiga toxin type 1. J. Clin. Microbiol..

[15] Paton J.C., Paton A.W. (1997). Instability of a Shiga toxin type 2 gene in *Enterobacter cloacae*. J. Clin. Microbiol..

[16] Kimura T., Tani S., Yi Y.M., Takeda T. (2001). Serum amyloid P component is the Shiga toxin 2-neutralizing factor in human blood. J. Biol. Chem..

[17] Marcato P., van der Helm K., Mulvey G.L., Armstrong G.D. (2003). Serum amyloid P component binding to Shiga toxin 2 requires both a subunit and B pentamer. Infect. Immun..

[18] Armstrong G.D., Mulvey G.L., Marcato P., Griener T.P., Kahan M.C., Tennent G.A., Sabin C.A., Chart H., Pepys M.B. (2006). Human serum amyloid P component protects against *Escherichia coli* O157:H7 Shiga toxin 2 *in vivo*: Therapeutic implications for hemolytic-uremic syndrome. J. Infect. Dis..

[19] Caprioli A., Luzzi I., Seganti L., Marchetti M., Karmali M.A., Clarke I., Boyd B., Karmali M.A., Goglio A. (1994). Frequency and nature of verocytotoxin 2 (VT2) neutralizing activity (NA) in human and animal sera. Recent Advances in Verocytotoxin-Producing Escherichia coli Infections.

[20] He X., Qi W., Quinones B., McMahon S., Cooley M., Mandrell R.E. (2011). Sensitive detection of Shiga toxin 2 and some of its variants in environmental samples by a novel immuno-PCR assay. Appl. Environ. Microbiol..

[21] Feng P.C., Jinneman K., Scheutz F., Monday S.R. (2011). Specificity of PCR and serological assays in the detection of *Escherichia coli* Shiga toxin subtypes. Appl. Environ. Microbiol..

[22] Willford J., Mills K., Goodridge L.D. (2009). Evaluation of three commercially available enzyme-linked immunosorbent assay kits for detection of Shiga toxin. J. Food Prot..

[23] Skinner C., Patfield S., Hernlem B.J., He X. (2015). New Stx2e Monoclonal Antibodies for Immunological Detection and Distinction of Stx2 Subtypes. PloS ONE.

[24] Alam S.I., Kumar B., Kamboj D.V. (2012). Multiplex detection of protein toxins using MALDI-TOF-TOF tandem mass spectrometry: Application in unambiguous toxin detection from bioaerosol. Anal. Chem..

[25] Conrady D.G., Flagler M.J., Friedmann D.R., van der Wielen B.D., Kovall R.A., Weiss A.A., Herr A.B. (2010). Molecular basis of differential B-pentamer stability of Shiga toxins 1 and 2. PloS ONE.

[26] Fagerquist C.K., Sultan O. (2010). Top-down proteomic identification of furin-cleaved alpha-subunit of Shiga toxin 2 from *Escherichia coli* O157:H7 using MALDI-TOF-TOF-MS/MS. J. Biomed. Biotechnol..

[27] Fagerquist C.K., Sultan O. (2011). Induction and identification of disulfide-intact and disulfide-reduced beta-subunit of Shiga toxin 2 from *Escherichia coli* O157:H7 using MALDI-TOF-TOF-MS/MS and top-down proteomics. Analyst.

[28] Kitova E.N., Daneshfar R., Marcato P., Mulvey G.L., Armstrong G., Klassen J.S. (2005). Stability of the homopentameric B subunits of Shiga toxins 1 and 2 in solution and the gas phase as revealed by nanoelectrospray fourier transform ion cyclotron resonance mass spectrometry. J. Am. Soc. Mass Spectrom..

[29] Kitova E.N., Kitov P.I., Bundle D.R., Klassen J.S. (2001). The observation of multivalent complexes of Shiga-like toxin with globotriaoside and the determination of their stoichiometry by nanoelectrospray fourier-transform ion cyclotron resonance mass spectrometry. Glycobiology.

[30] Kitova E.N., Kitov P.I., Paszkiewicz E., Kim J., Mulvey G.L., Armstrong G.D., Bundle D.R., Klassen J.S. (2007). Affinities of Shiga toxins 1 and 2 for univalent and oligovalent PK-trisaccharide analogs measured by electrospray ionization mass spectrometry. Glycobiology.

[31] Kitova E.N., Mulvey G.L., Dingle T., Sinelnikov I., Wee S., Griener T.P., Armstrong G.D., Klassen J.S. (2009). Assembly and stability of the Shiga toxins investigated by electrospray ionization mass spectrometry. Biochemistry.

[32] Kondo F., Kobayashi S., Matsumoto M., Yamada S., Saito M., Suzuki Y., Ishikawa N., Nakanishi T., Shimizu A. (1997). Analysis of Vero toxins 1 and 2 by high-performance liquid chromatography/electrospray ionization mass spectrometry. J. Mass Spectrom..

[33] Kondo F., Saito H., Hayashi R., Onda H., Kobayashi S., Matsumoto M., Suzuki M., Ito Y., Oka H., Nakanishi T. (2003). Identification of Shiga toxins in Shiga toxin-producing *Escherichia coli* using immunoprecipitation and high-performance liquid chromatography-electrospray ionization mass spectrometry. Analyst.

[34] Meisen I., Friedrich A.W., Karch H., Witting U., Peter-Katalinic J., Muthing J. (2005). Application of combined high-performance thin-layer chromatography immunostaining and nanoelectrospray ionization quadrupole time-of-flight tandem mass spectrometry to the structural characterization of high- and low-affinity binding ligands of Shiga toxin 1. Rapid Commun. Mass Spectrom..

[35] Silva C.J., Erickson-Beltran M.L., Skinner C.B., Dynin I., Hui C., Patfield S.A., Carter J.M., He X. (2014). Safe and effective means of detecting and quantitating Shiga-like toxins in attomole amounts. Anal. Chem..

[36] Domon B., Aebersold R. (2006). Mass spectrometry and protein analysis. Science.

[37] Pan S., Aebersold R., Chen R., Rush J., Goodlett D.R., McIntosh M.W., Zhang J., Brentnall T.A. (2009). Mass spectrometry based targeted protein quantification: Methods and applications. J. Proteome Res..

[38] Picotti P., Aebersold R. (2012). Selected reaction monitoring-based proteomics: Workflows, potential, pitfalls and future directions. Nat. Methods.

[39] ExPASy. http://www.expasy.org/tools/.

[40] Silva C.J., Dynin I., Erickson M.L., Requena J.R., Balachandran A., Hui C., Onisko B.C., Carter J.M. (2013). Oxidation of Methionine 216 in Sheep and Elk Prion Protein Is Highly Dependent upon the Amino Acid at Position 218 but Is not Important for Prion Propagation. Biochemistry.

[41] Roepstorff P., Fohlman J. (1984). Proposal for a common nomenclature for sequence ions in mass spectra of peptides. Biomed. Mass Spectrom..

[42] Skinner C., McMahon S., Rasooly R., Carter J.M., He X. (2013). Purification and characterization of Shiga toxin 2f, an immunologically unrelated subtype of Shiga toxin 2. PLoS ONE.

[43] He X., Patfield S., Hnasko R., Rasooly R., Mandrell R.E. (2013). A polyclonal antibody based immunoassay detects seven subtypes of Shiga toxin 2 produced by *Escherichia coli* in human and environmental samples. PLoS ONE.

[44] Onisko B., Dynin I., Requena J.R., Silva C.J., Erickson M., Carter J.M. (2007). Mass spectrometric detection of attomole amounts of the prion protein by nanoLC/MS/MS. J. Am. Soc. Mass Spectrom..

[45] Sambrook J., Fritsch E.F., Maniatis T. (1989). Molecular Cloning: A Laboratory Manual.

