# Supplementary material for: Mass Spectrometry-Based Method of Detecting and Distinguishing Type 1 and Type 2 Shiga-Like Toxins in Human Serum
Source: Toxins (Basel). 2015 Dec 2;7(12):5236–53. doi: 10.3390/toxins7124875 (PMC4690125; doi:10.3390/toxins7124875)
Supplement: Supplementary file 1 [file toxins-07-04875-s001.pdf]

Supplementary Materials: Mass Spectrometry-Based Method of Detecting and Distinguishing Type 1 and Type 2 Shiga-Like Toxins in Human Serum

Table S1. A table of instrument parameters, collision energy (CE), declustering potential (DP), entrance potential (EP), and collision exit potential (CXP) for the listed peptides from Stx1 and Stx2 subtypes.

Specificity	Peptide	Ion	^{14}N or ^{15}N	Q1 Mass (Da)	Q3 Mass (Da)	Dwell (msec)	DP	EP	CE	CXP
Stx2	IEFSK	y_3	^{14}N	312.2	381.1	50	70	10.2	17	5.8
		y_4		312.2	510.1	50	70	10.2	17	8
Stx2	IEFSK	y_3	^{15}N	315.2	385.2	50	70	10.2	17	5.8
		y_4		315.2	515.3	50	70	10.2	17	8
Stx1	VEYTK	y_3	^{14}N	320.4	411.1	50	80.2	10.6	17.7	5.9
		y_4		320.4	540.1	50	80.2	10.6	18	9.9
Stx1	VEYTK	y_3	^{15}N	323.2	415.2	50	80.2	10.6	17.7	5.9
		y_4		323.2	545.3	50	80.2	10.6	18	9.9
Stx1a	ELFTNR	a_2	^{14}N	390.21	215.14	50	65	10.6	26.5	4.3
		y_2		390.21	289.16	50	65	10.6	18.4	7.1
		y_4		390.21	537.28	50	65	10.6	22.5	8.5
Stx1a	ELFTNR	a_2	^{15}N	395.19	217.13	50	65	10.6	26.5	4.3
		y_2		395.19	295.14	50	65	10.6	18.4	7.1
		y_4		395.19	545.25	50	65	10.6	22.5	8.5
Stx1e	ELYTTR	y_2	^{14}N	391.71	276.17	50	67	8.77	17.7	15
		y_3		391.71	377.21	50	67	8.77	20	15
		y_4		391.71	540.28	50	67	8.77	22	15
Stx1e	ELYTTR	y_2	^{15}N	396.19	281.15	50	67	8.77	17.7	15
		y_3		396.19	383.2	50	67	8.77	20	15
		y_4		396.19	547.26	50	67	8.77	22	15
Stx2a, c, d	EYWTSR	y_3	^{14}N	421.2	363.1	50	75	10.4	22	10.2
		y_4		421.2	549.1	50	75	10.4	23	8.4
Stx2a, c, d	EYWTSR	y_3	^{15}N	426.3	369.2	50	75	10.4	22	10.2
		y_4		426.3	557.3	50	75	10.4	23	8.4
Stx2b, e, f, g	EYWTNR	y_3	^{14}N	434.9	390.1	50	75	10.1	22	5.3
		y_4		434.9	576.1	50	75	10.1	24	9.8
Stx2b, e ,f, g	EYWTNR	y_3	^{15}N	440.2	397.2	50	75	10.1	22	5.3
		y_4		440.2	585.3	50	75	10.1	24	9.8
Stx2g	YNGDNTFTVK	b_2	^{14}N	579.8	278.1	50	114	11	27.6	15
		y_6		579.8	709.4	50	114	11	27.6	15
		y_7		579.8	824.4	50	114	11	27.6	15
		y_8		579.8	881.5	50	114	11	26.3	15
Stx2g	YNGDNTFTVK	b_2	^{15}N	586.3	281.1	50	114	9.13	29	15
		y_6		586.3	717.4	50	90	9.13	29	15
		y_7		586.3	833.4	50	90	9.13	29	15
		y_8		586.3	891.4	50	90	9.13	27.5	15

Table S1. *Cont.*

Specificity	Peptide	Ion	^{14}N or ^{15}N	Q1 Mass (Da)	Q3 Mass (Da)	Dwell (msec)	DP	EP	CE	CXP
Stx1-E29	YNDDDTFTAK	b_2	^{14}N	595.25	278.11	50	90	9.13	29	15
		y_6		595.25	682.34	50	90	9.13	29	15
		y_7		595.25	797.37	50	90	9.13	29	15
		y_8		595.25	912.4	50	90	9.13	27.5	15
Stx1-E29	YNDDDTFTAK	b_2	^{15}N	601.24	281.11	50	90	9.13	29	15
		y_6		601.24	689.32	50	90	9.13	29	15
		y_7		601.24	805.34	50	90	9.13	29	15
		y_8		601.24	921.37	50	90	9.13	29	15
Stx1a, e	YNDDDTFTVK	b_2	^{14}N	609.3	278.1	50	125	11	29.4	15
		y_6		609.3	710.4	50	125	11	29.4	15
		y_7		609.3	825.4	50	125	11	29.4	15
		y_8		609.3	940.4	50	125	11	28.7	15
Stx1a, e	YNDDDTFTVK	b_2	^{15}N	615.3	281.1	50	125	11	29.4	15
		y_6		615.3	717.4	50	125	11	29.4	15
		y_7		615.3	833.4	50	125	11	29.4	15
		y_8		615.3	949.4	50	125	11	28.7	15
Stx2b, c, d	YNENDTFTVK	b_2	^{14}N	615.8	278.2	50	115	11	29.2	15
Stx2e	YNEDNTFTVK	y_8		615.8	953.4	50	115	11	28	15
Stx2a, f	YNEDDTFTVK	b_2	^{15}N	616.3	278.1	50	115	11	29.2	15
		y_8		616.3	954.4	50	115	11	28	15
Stx2b, c, d, e, f	YNEDDTFTVK	b_2	^{15}N	622.3	281.1	50	115	11	29.2	15
	YNENDTFTVK<br>YNEDNTFTVK	y_8		622.3	963.4	50	115	11	28	15

Table S2. Retention times of peptides used in this study. The retention times are derived from the MRM analysis of the trypsin digest of the ^{15}N-labeled Stx-IS protein.

Peptide	Retention Time (min)
YNDDDTFTVK	11.51
YNDDDSFTVK	11.33
YNDDDTFTAK	10.78
EYWTNR	11.28
EYWTSR	11.28
ELYTTR	10.52
ELFTNR	11.60
IEFSK	11.00

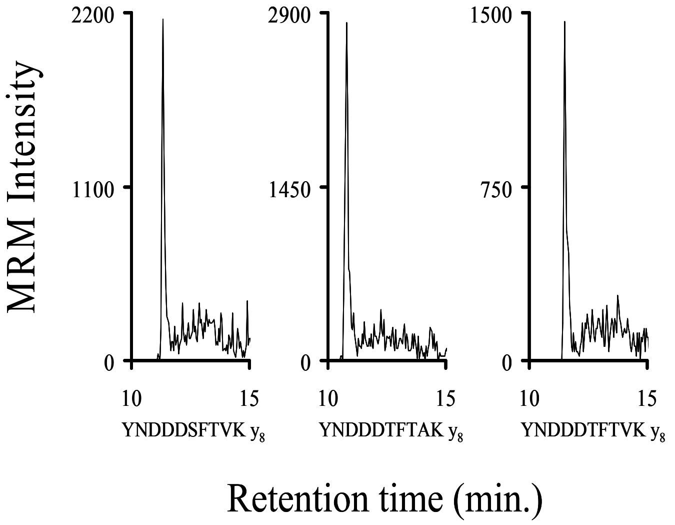

Figure S1. MRM signal intensities for the y_8 ions from the ^{15}N-labeled analyte peptides for Stx1. The signals from the peptides are derived from the trypsin digest of the ^{15}N-labeled Stx internal standard protein.

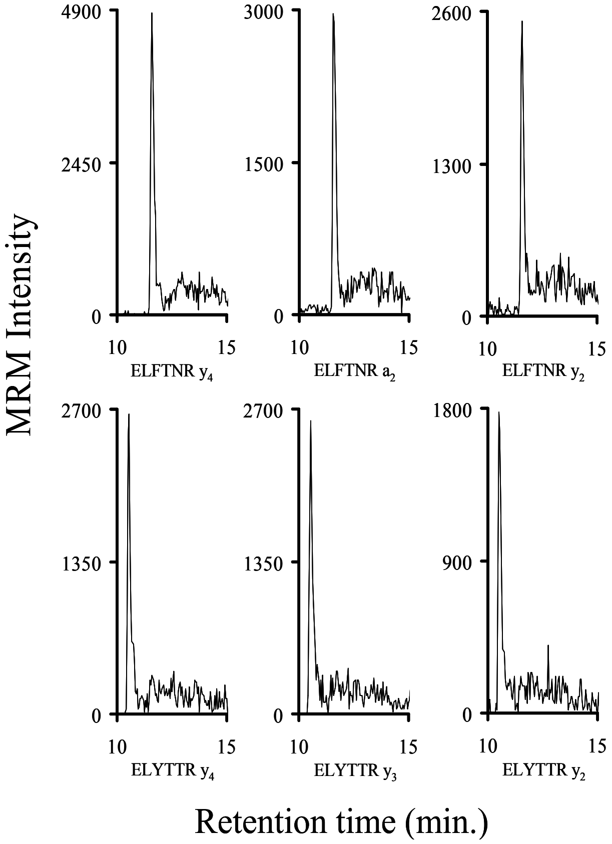

Figure S2. MRM signal intensities for the three most intense ions from ELFTNR (Stx1a) and ELYTTR (Stx1e). The signals from the peptides are derived from the trypsin digest of the ^{15}N-labeled Stx internal standard protein.

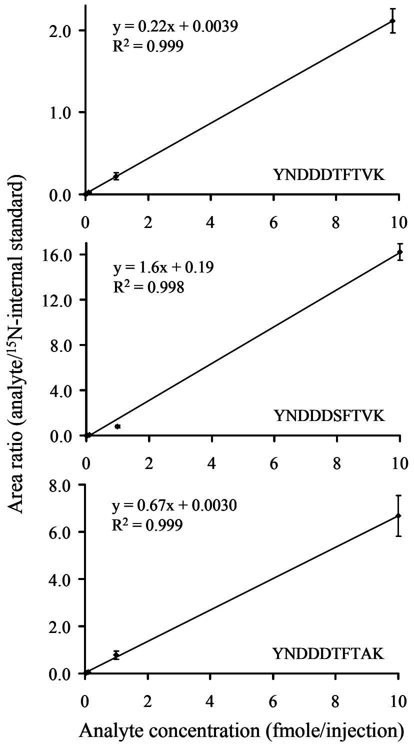

Analyte Conc. (amol/inj)	Area ratio
9,800	2 ± 0.1
981	0.2 ± 0.04
98	0.03 ± 0.07
49	0.01 ± 0.03
20	0.008 ± 0.003

Analyte Conc. (amol/inj)	Area ratio
10,000	16 ± 0.7
1,000	0.8 ± 0.1
100	0.06 ± 0.02
50	0.02 ± 0.02
20	0.01 ± 0.08

Analyte Conc. (amol/inj)	Area ratio
10,000	6.7 ± 0.8
1,000	0.8 ± 0.2
100	0.05 ± 0.01
50	0.04 ± 0.02
20	0.000

Figure S3. Calibration curves used to determine the empirical relationship between the concentration (~20 amole–10 fmole) of the analyte peptide and the respective ^{15}N-labeled internal standards. All analyte peptides are from Stx1 subtypes (YNDDDTFTVK, YNDDDSFTVK, and YNDDDTFTAK). All concentrations are reported as the mean (±standard deviation) of four injections. The data used to prepare a given curve is shown to the right of that curve.

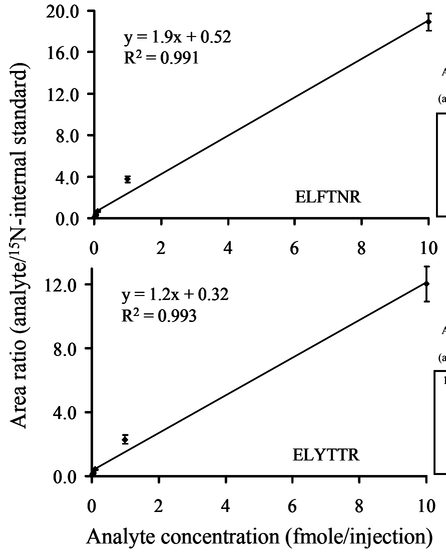

Analyte Conc. (amol/inj)	Area ratio
10,000	19 ± 0.8
1,000	3.7 ± 0.3
100	0.7 ± 0.08
50	0.3 ± 0.04
20	0.1 ± 0.03

Analyte Conc. (amol/inj)	Area ratio
10,000	12 ± 1
1,000	2.3 ± 0.3
100	0.4 ± 0.02
50	0.2 ± 0.003
20	0.1 ± 0.003

Figure S4. Calibration curves used to determine the empirical relationship between the concentration (~10 amole–10 fmole) of the ELFTNR (Stx1a) and ELYTTR (Stx1e) tryptic peptides and their respective ^{15}N-labeled internal standards. All concentrations are reported as the mean (±standard deviation) of four injections. The data used to prepare a given curve is shown to the right of that curve.

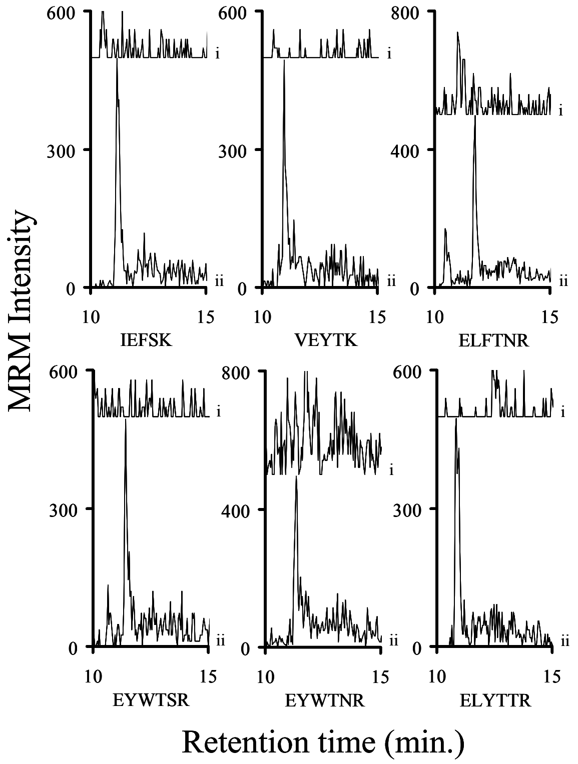

Figure S5. Chromatograms of the MRM signal intensities derived from the tryptic digests of human serum showing no interference with the y_3 or the y_4 ion of the IEFSK, VEYTK, ELFTNR, EYWTSR, EYWTNR, and ELYTTR peptides from Stx1 and Stx2 subtypes. The signals corresponding to the y_3 or y_4 ion of tryptic peptides from the Shiga-like toxins Stx2 (IEFSK; y_4); Stx1 (VEYTK; y_4); Stx1a (ELFTNR; y_4); Stx2a, Stx2b, and Stx2c, (EYWTSR; y_4); or Stx2d, Stx2e, Stx2f, and Stx2g (EYWTNR; y_3); or Stx1e (ELYTTR; y_4) are shown in each graph (i); Each sample was spiked with the corresponding ^{15}N-labeled internal standard (ii); The signals for the ions from the corresponding ^{15}N-labeled internal standards (ii) are normalized to 500. The other signals are not normalized. The graphs are offset for clarity.

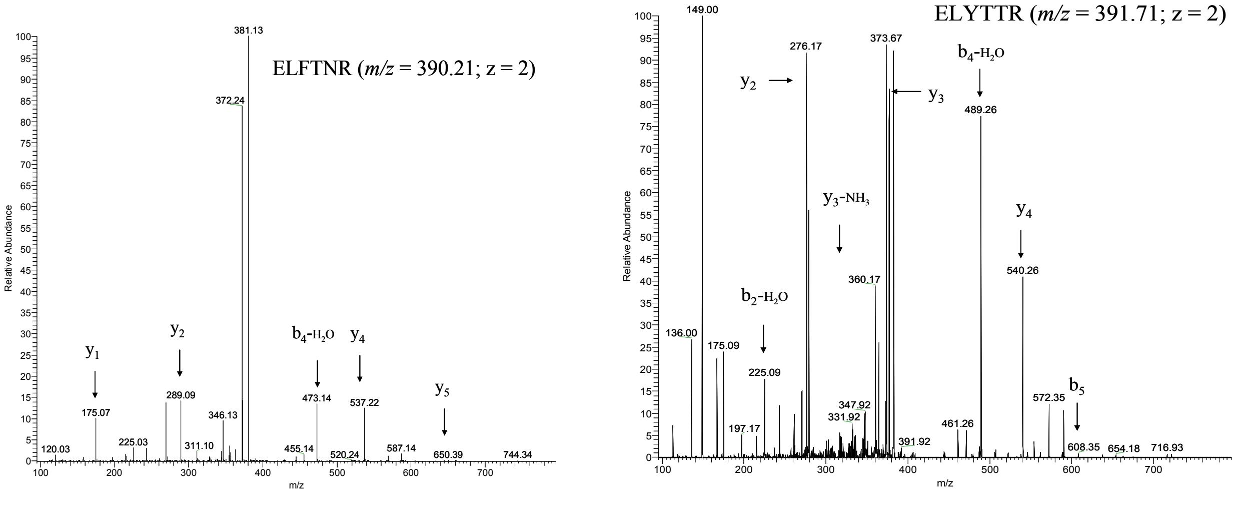

(**a**) (**b**)

Figure S6. *Cont.*

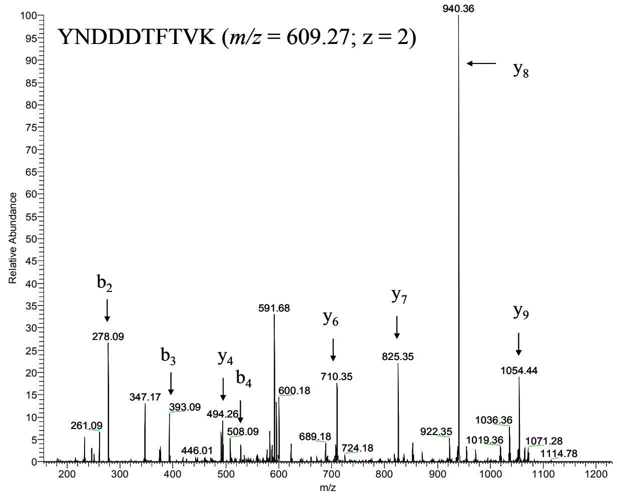

(**c**)

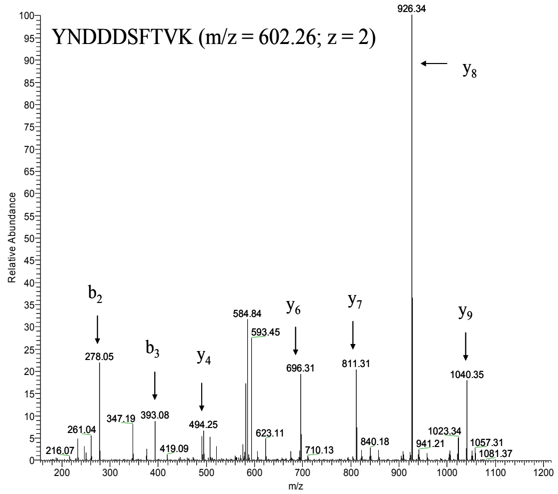

(**d**)

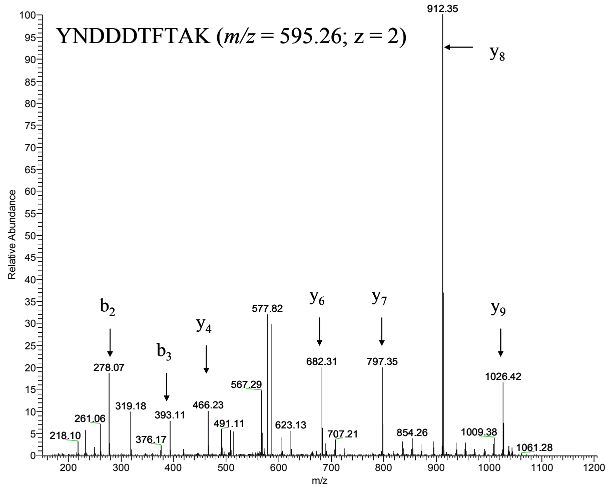

(**e**)

Figure S6. MS-MS fragmentation mass spectra of the synthetic peptides ELFTNR (**a**), ELYTTR (**b**), YNDDDTFTVK (**c**), YNDDDSFTVK (**d**), and YNDDDTFTAK (**e**). Samples were run on an Orbitrap elite instrument using full scan MS followed by MS-MS on the ten most intense ions. The difference between the calculated monoisotopic mass and the observed mass was 10 ppm or less for all five peptides.
